# Supplementary material for: Rare Occurrence of Aristolochic Acid Mutational Signatures in Oro-Gastrointestinal Tract Cancers
Source: Cancers (Basel). 2022 Jan 24;14(3):576. doi: 10.3390/cancers14030576 (PMC8833562; doi:10.3390/cancers14030576)
Supplement: Supplementary file 1 [file cancers-14-00576-s001.zip › cancers-1520411-supplementary.pdf]

cancers MDPI

Supplementary Materials: Rare occurrence of Aristolochic Acid Mutational Signatures in Oro-Gastrointestinal Tract Cancers

Abner Herbert Lim, Jason Yongsheng Chan, Ming-Chin Yu, Tsung-Han Wu, Jing Han Hong, Cedric Chuan-Young Ng, Zhen Jie Low, Wei Liu, Rajasegaran Vikneswari, Pin-Cheng Sung, Wen-Lang Fang, Bin Tean The * and Sen-Yung Hsieh *

A.

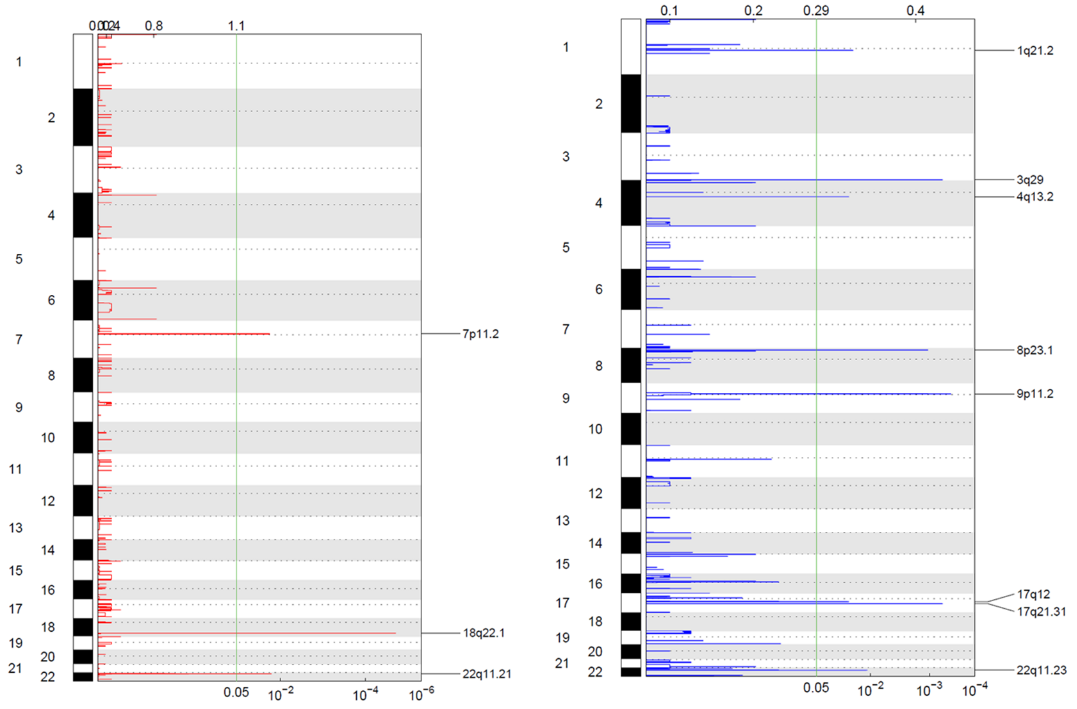

B.

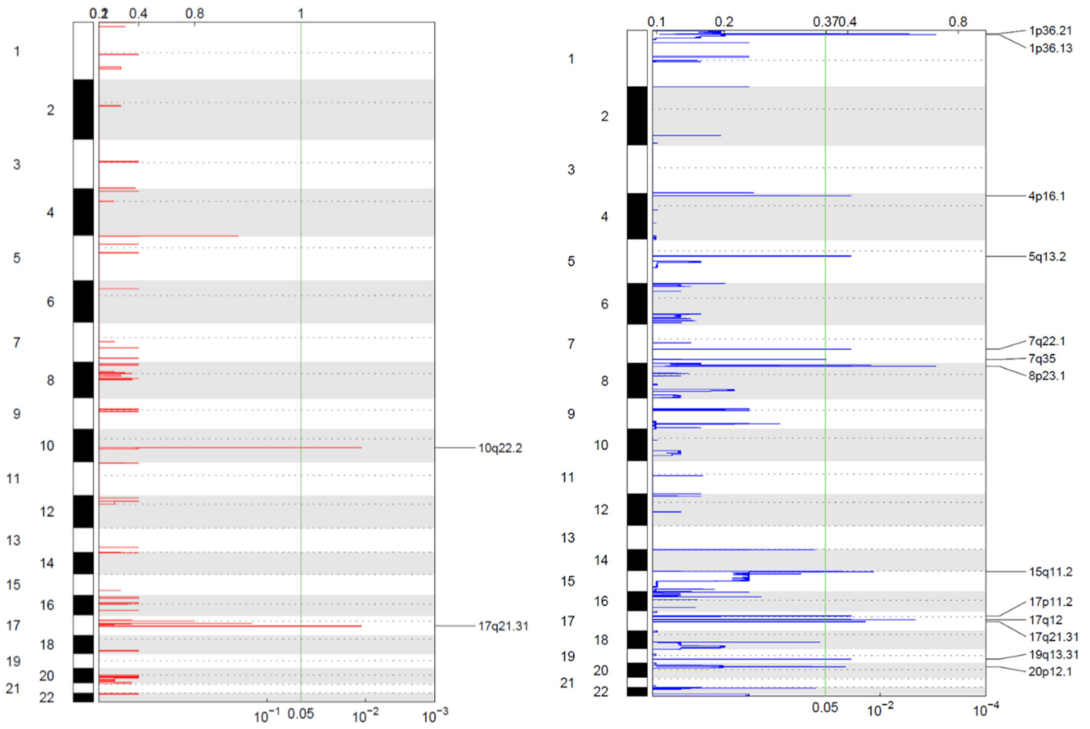
10q22.2
17q21.31
1p36.21
1p36.13
4p16.1
5q13.2
7q22.1
7q35
8p23.1
15q11.2
17p11.2
17q12
17q21.31
19q13.31
20p12.1

C.

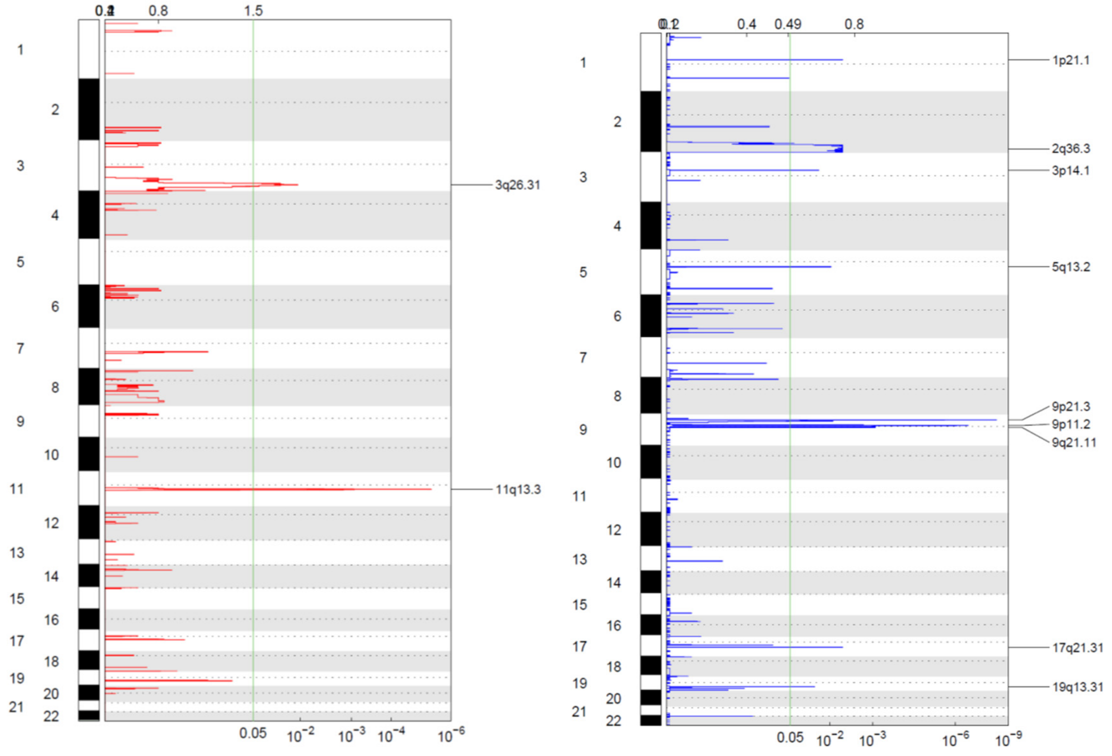
3q26.31
11q13.3
1p21.1
2q36.3
3p14.1
5q13.2
9p21.3
9p11.2
9q21.11
17q21.31
19q13.31

D.

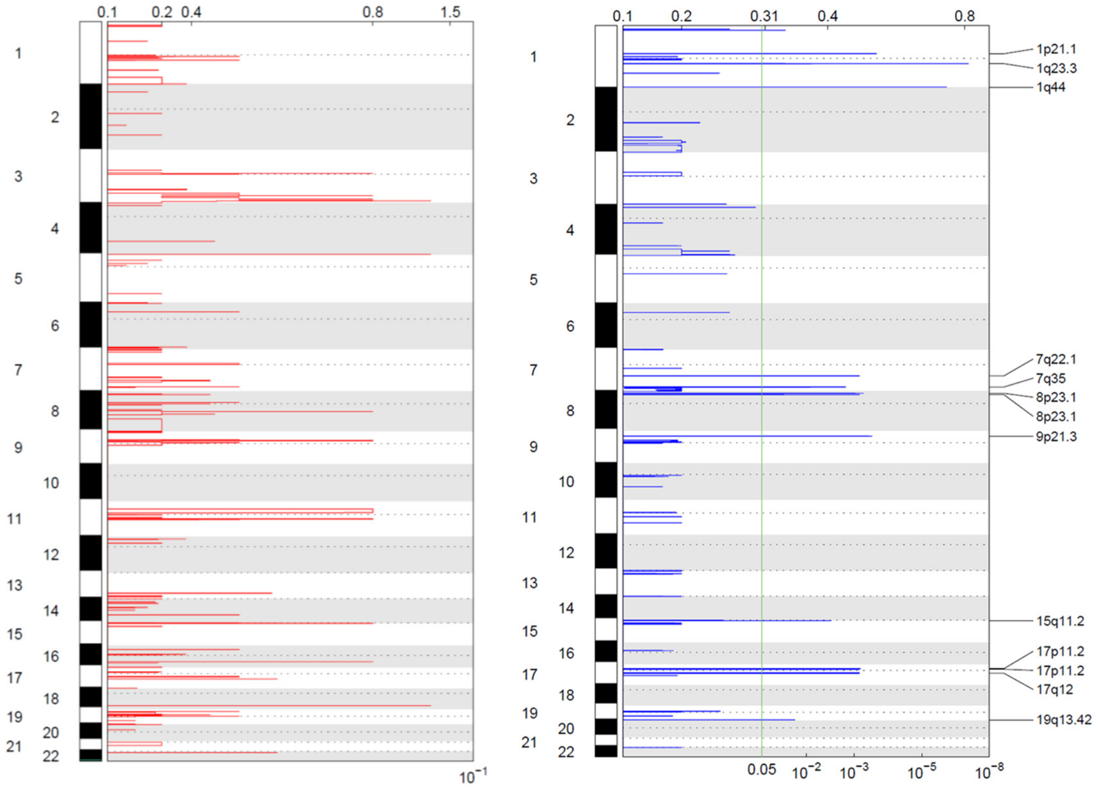

Figure S1. GISTIC results: Regions of significant copy number aberrations. (A) GA (B) CA (C) ESCC (D) OSCC.

A.

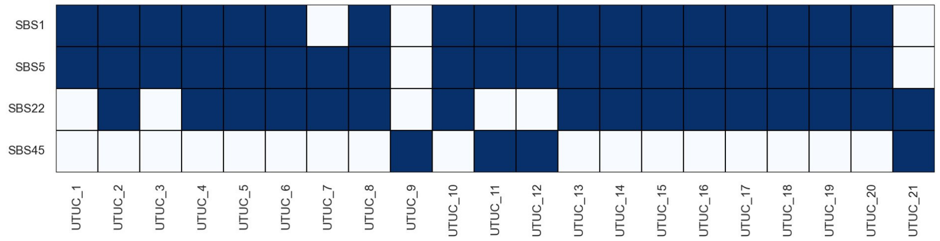

B.

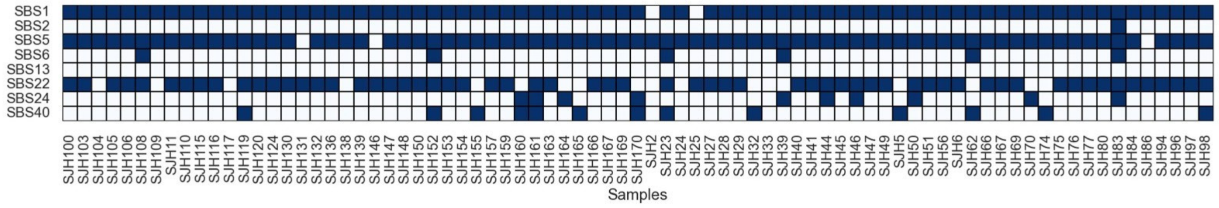

C.

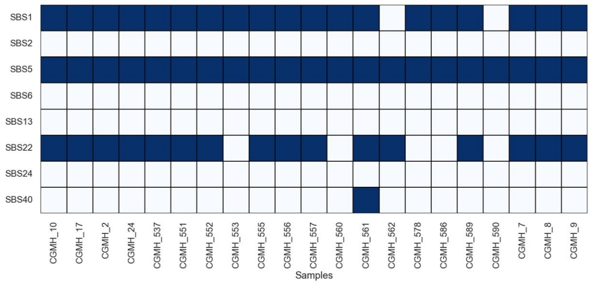

Figure S2. Mutational signatures in UTUC and HCC. (A) Mutational signatures present in UTUC (B&C) Mutational signatures present in HCC. Both retrospective studies show high proportion of AA exposure (SBS22) in cancers of liver and urinary tract.

Table S1. Previously reported publications on AA-signatures in various cancers.

UTUC	TP53 Amplicon with AL-DNA Adducts	148	38 (AL-DNA Adduct Positive with TP53 A->T Transversions)	Chen et al, 2012 [44]
AA-UTUC	WES	9	9	Poon et al, 2013 [23]
UTUC	WES	19	17	Hoang et al, 2013 [25]
BC	WGS	13	3	Poon et al, 2015 [21]
ccRCC	WES	10	6	Hoang et al, 2016 [22]
HCC	WES	98	76	Ng et al, 2017 [20]
UTUC	WGS	90	27	Lu et al, 2020 [24]
